# Integrated Molecular and Phenotypic Profiling of Circulating Tumor Cells in Gastric Cancer: A Translational Perspective

**DOI:** 10.1096/fba.2025-00314

**Published:** 2026-08-19

**Authors:** Mohammad Reza Eskandarion, Sharareh Eskandarieh, Abbas Shakoori Farahani, Farideh Ghanbari Mardasi, Habibollah Mahmoodzadeh, Kamran Roudini, Farhad Shahi, Mohammad Ali Oghabian, Mohammad Taher, Reza Shirkoohi

**Affiliations:** ^1^ Cancer Research Center, Cancer Institute, Imam Khomeini Hospital Complex Tehran University of Medical Sciences Tehran Iran; ^2^ Multiple Sclerosis Research Center, Neuroscience Institute Tehran University of Medical Sciences Tehran Iran; ^3^ Medical Genetics Ward, Imam Khomeini Hospital Complex Tehran University of Medical Sciences Tehran Iran; ^4^ Department of Medical Genetics School of Medicine Tehran University of Medical Sciences Tehran Iran; ^5^ Department of Surgery, Cancer Research Center, Cancer Institute, Imam Khomeini Hospital Complex Tehran University of Medical Sciences Tehran Iran; ^6^ Department of Internal Medicine, Hematology and Medical Oncology Ward, Cancer Research Center, Imam Khomeini Hospital Complex Tehran University of Medical Sciences, Cancer Institute Tehran Iran; ^7^ Department of Medical Oncology, Cancer Research Center, Cancer Institute, Imam Khomeini Hospital Complex Tehran University of Medical Sciences Tehran Iran; ^8^ Medical Physics Department, Faculty of Medicine Tehran University of Medical Sciences Tehran Iran; ^9^ Division of Gastroenterology and Hepatology, Imam Khomeini Hospital Complex Tehran University of Medical Sciences Tehran Iran; ^10^ Liver Transplantation Research Center Tehran University of Medical Sciences Tehran Iran

**Keywords:** circulating tumor cells, expression analysis, gastric cancer, magnetic‐activated cell sorting, miR‐29b

## Abstract

Gastric cancer often presents at advanced stages, limiting treatment outcomes. Circulating tumor cells (CTCs), particularly those undergoing epithelial–mesenchymal transition (EMT), have emerged as promising biomarkers for real‐time disease monitoring. Using magnetic‐activated cell sorting (MACS), we enriched both epithelial (EpCAM^+^) and mesenchymal (CD90^+^) CTCs and evaluated the expression of the tumor‐suppressive microRNA miR‐29b in CTCs of gastric cancer patients. This study aimed to explore the clinical relevance of perioperative CTC phenotypes and miR‐29b as potential diagnostic and prognostic markers. We conducted a multi‐phase study integrating systematic review, bioinformatics, and experimental analyses to investigate CTC phenotypes and ECM‐associated gene expression in GC. Peripheral blood samples were collected from 30 treatment‐naïve gastric cancer patients at two time points: before initiation of therapy and 1 month after curative‐intent gastrectomy. Circulating tumor cells (CTCs) were isolated using CD45‐negative magnetic‐activated cell sorting (MACS). Immunofluorescence staining was performed to phenotype CTCs based on EpCAM (epithelial) and CD90 (mesenchymal) expression. Total RNA was extracted from tumor tissues and isolated CTCs to evaluate ECM‐related genes and miR‐29b expression using quantitative real‐time PCR. Bioinformatics‐guided gene selection and statistical analysis were used to assess diagnostic and prognostic relevance. CTCs were detected in 83.3% (25/30) of gastric cancer patients prior to treatment. Mesenchymal CTCs (CD90^+^) were markedly more frequent than epithelial CTCs (EpCAM^+^), with mean baseline counts of 14.07 versus 0.5 cells per 10 mL of blood, respectively. Following gastrectomy and chemotherapy, mesenchymal CTCs significantly declined (mean: 1.18; *p* = 0.0064), while epithelial CTCs were nearly undetectable (*p* = 0.0246). Based on perioperative CTC dynamics, patients were stratified into three risk groups: high‐risk (16%), intermediate‐risk (68%), and low‐risk (16%). All observed deaths occurred in the high‐risk group during 14 months of follow‐up, with Kaplan–Meier analysis showing significantly lower survival in this group (*p* = 0.0107). Expression analysis revealed significant downregulation of miR‐29b in tumor tissues (fold change = 0.5163; *p* = 0.0136) and in CTCs (fold change = 0.2049; *p* < 0.0001) compared to healthy controls. Although miR‐29b levels were broadly downregulated in gastric cancer patients, no significant differences were observed among clinicopathological subgroups within the patient cohort. ROC analysis showed strong diagnostic performance of miR‐29b in tissue (AUC = 0.883, 95% CI: 0.798–0.969), with 96.7% sensitivity and 70% specificity. CD90 serves as a sensitive mesenchymal marker for detecting CTCs that lack epithelial features, enabling more comprehensive identification of aggressive CTC phenotypes in gastric cancer. Moreover, the perioperative dynamics of CTCs—especially postoperative increases—provide a valuable tool for patient risk stratification and survival prediction. Importantly, our findings suggest that miR‐29b expression in CTCs, together with perioperative CTC dynamics, may serve as a non‐invasive biomarker for monitoring disease progression and recurrence in gastric cancer.

## Graphical abstract

Peripheral blood samples from gastric cancer patients were analyzed before and after curative‐intent treatment using CD45‐negative MACS enrichment. Mesenchymal CD90^+^ circulating tumor cells predominated over epithelial EpCAM^+^ cells and declined significantly following treatment. Concurrent downregulation of miR‐29b was observed in both tumor tissues and isolated circulating tumor cells. Integration of perioperative circulating tumor cell dynamics enabled risk stratification and identified patients with significantly poorer overall survival, supporting the clinical utility of liquid biopsy–based monitoring in gastric cancer.

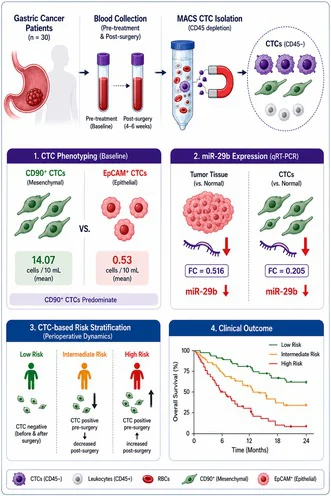

## 1 Introduction

Gastric cancer (GC) is the fifth most commonly diagnosed malignancy and the third leading cause of cancer‐related mortality worldwide, with over 1 million new cases and nearly 770,000 deaths reported annually [1]. Despite advances in therapy, the prognosis for GC remains poor due to late‐stage diagnosis and high rates of recurrence [2]. Therefore, there is an urgent need for more accurate, minimally invasive methods for early detection, prognosis, and treatment monitoring.

Circulating tumor cells (CTCs), which detach from primary tumors and enter the bloodstream, have emerged as promising biomarkers in solid tumors. These cells reflect the dynamic biological state of the tumor and provide a real‐time snapshot of disease progression and therapeutic response [3]. In GC, CTC detection is of particular importance given the tumor's high heterogeneity and late presentation.

Recent large‐scale evidence further highlights the variability of CTC detection in gastric cancer depending on the methodology applied. In our recent systematic review and meta‐analysis including 45 studies and 3342 patients, the overall pooled prevalence of CTCs in gastric cancer was 69.37% (95% CI: 60.27–77.78), with substantial heterogeneity across platforms. Detection rates varied by clinical stage, reflecting disease burden, with pooled CTC prevalence of 44.48% in early‐stage disease and 56.17% in advanced‐stage disease. Importantly, subgroup analyses demonstrated that both epithelial marker–based and mesenchymal marker–based approaches were associated with significant prognostic value for overall survival (HR = 2.75, 95% CI: 2.34–3.24) and progression‐free survival (HR = 2.78, 95% CI: 2.01–3.85). Furthermore, risk‐difference analyses suggested that mesenchymal marker–based strategies and CellSearch platforms may exhibit differential detection performance depending on sampling time and tumor stage. These findings underscore the methodological heterogeneity in CTC analysis and the need for complementary marker integration to improve biological characterization and clinical stratification [4]. However, despite extensive meta‐analytic evidence regarding CTC detection platforms, relatively few studies have integrated mesenchymal‐specific markers with dynamic perioperative risk modeling—an unmet need that forms the rationale for the present study.

CTCs exhibit considerable phenotypic diversity, particularly with respect to epithelial–mesenchymal transition (EMT). Epithelial markers such as EpCAM are commonly used for CTC enrichment, while mesenchymal markers like CD90 have been associated with enhanced migratory capacity and poor prognosis [5]. Thus, dual‐phenotypic characterization of CTCs offers more comprehensive clinical insight.

Magnetic‐Activated Cell Sorting (MACS), based on surface marker expression, is a widely used technique for isolating both epithelial and mesenchymal CTCs with high efficiency and cell viability [6]. It provides a feasible and scalable approach to monitor CTCs before and after treatment interventions such as surgery or chemotherapy.

To address this unmet need, we integrated MACS‐based isolation of epithelial and mesenchymal CTCs with downstream molecular profiling approaches in gastric cancer [4].

In the next phase, a bioinformatics study was conducted to identify genes and pathways associated with extracellular matrix (ECM) remodeling in gastric cancer. Given the pivotal role of the ECM in metastasis, particular attention was given to ECM‐related genes and regulators, including microRNAs. The analysis identified several ECM‐related genes and **miR‐29b**, a known tumor suppressor involved in ECM regulation, as promising candidates for experimental validation [7].

Following this, the expression levels of selected ECM‐related genes and miR‐29b were assessed in tumor tissues of gastric cancer patients. Based on these findings, miR‐29b was prioritized for further evaluation in circulating tumor cells (CTCs) due to its potential as a non‐invasive biomarker for diagnosis and prognosis in GC.

This multi‐phase study aimed to [1] isolate and phenotype CTCs using MACS before treatment and after gastrectomy, [2] assess ECM‐related gene expression in tumor tissues, and [3] investigate miR‐29b expression in CTCs to determine its potential as a clinically relevant biomarker in gastric cancer management.

## 2 Materials and Methods

### 2.1 Study Design and Overview

**FIGURE 1:**
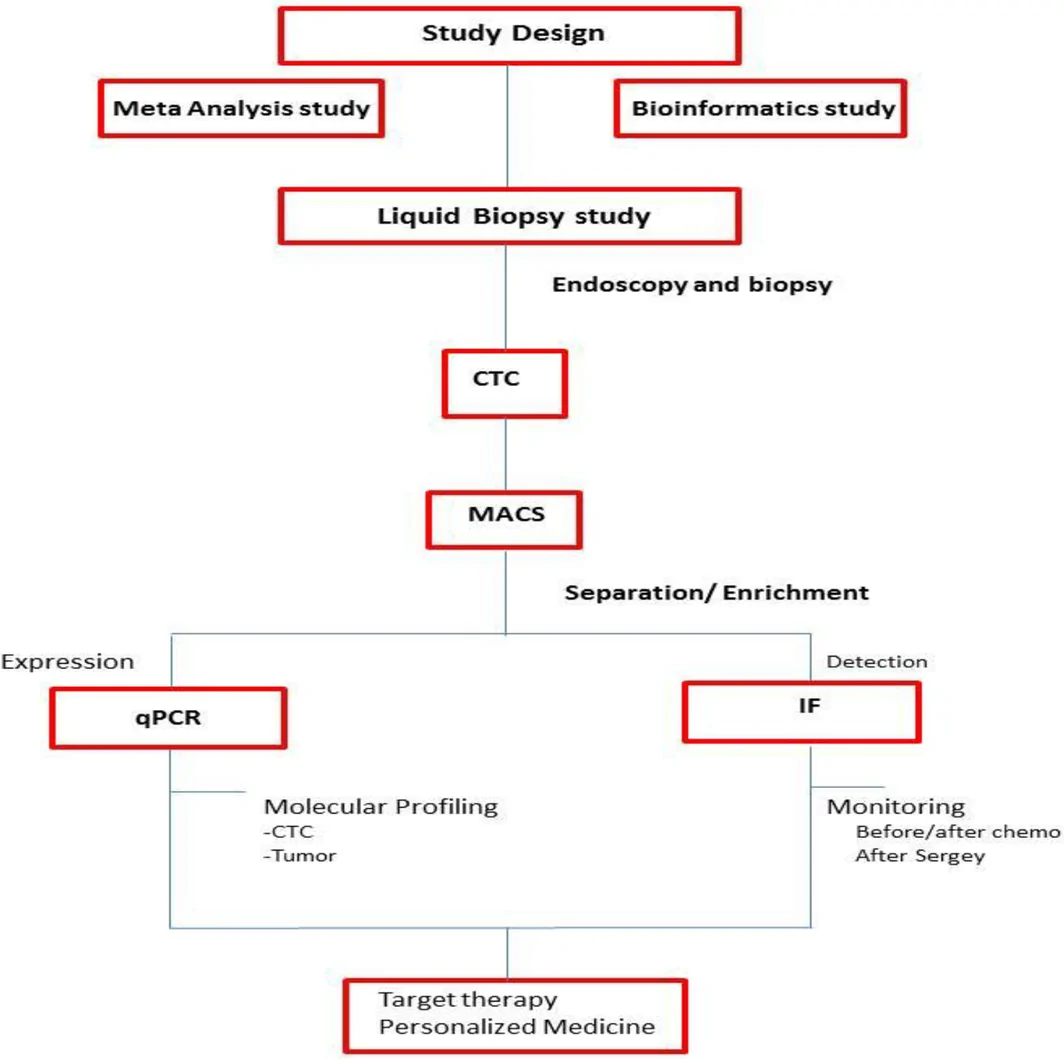
Schematic overview of the study workflow.

This study was conducted in four sequential phases to investigate the potential role of circulating tumor cells (CTCs) and ECM‐related gene expression in gastric cancer (GC) (see Figure 1 for the schematic overview):

1. **CTC isolation and enumeration**: Peripheral blood samples were collected from GC patients before treatment and 1 month after gastrectomy. CTCs were isolated and enumerated using the MACS platform.
2. **Experimental validation**: Gene expression levels of selected ECM components were validated in gastric tumor tissues, and miRNA expression was subsequently evaluated in isolated CTCs using quantitative PCR.

### 2.2 Study Population

This study enrolled 30 patients with histopathologically confirmed gastric adenocarcinoma who were referred to the Cancer Institute of Imam Khomeini Hospital, Tehran. Eligible participants included treatment‐naïve individuals scheduled for curative‐intent gastrectomy. Patients with a history of chemotherapy, radiotherapy, or other malignancies were excluded. Blood samples were collected at two time points: prior to initiation of any systemic therapy and approximately 1 week after surgical resection. Informed consent was obtained from all participants, and the study protocol was approved by the institutional ethics committee (Ethics Code: IR.TUMS.IKHC.REC.1401.196). Thirty healthy individuals with no history of malignancy were recruited as controls for molecular expression comparison.

#### 2.2.1 Clinical Staging and Nodal Assessment

All patients underwent clinical staging prior to surgery based on the 8th edition of the AJCC TNM classification system. The staging work‐up included contrast‐enhanced computed tomography (CT) of the chest, abdomen, and pelvis in all cases. Endoscopic ultrasound (EUS) was performed in all of the patients when technically feasible and available, particularly to assess depth of tumor invasion (T stage) and local nodal status. PET/CT was used selectively for patients with suspected distant disease or equivocal findings on CT.

Lymph nodes were considered radiologically positive when they exhibited a short‐axis diameter ≥ 8 mm (perigastric) or ≥ 10 mm (extra‐regional), or when showing suspicious morphological features such as round shape, central necrosis, or loss of fatty hilum. When EUS data were available, hypoechoic, round nodes > 5 mm were also regarded as suspicious. Clinical N stage (cN) was assigned accordingly, integrating all imaging findings and multidisciplinary review.

For statistical comparisons, patients were categorized into N0–N1 (limited nodal involvement) and N2–N3 (advanced nodal disease) groups. This grouping reflects a clinically relevant distinction in gastric cancer prognosis and therapeutic decision‐making, and it also provided adequate sample sizes for robust statistical comparison. A sensitivity analysis using a binary categorization (N0 vs. N+) was also performed to confirm the robustness of findings.

### 2.3 Blood Sample Collection and CTC Isolation

Peripheral blood mononuclear cells (PBMCs) were isolated from 10 mL of peripheral blood using Ficoll‐Paque density gradient centrifugation. Circulating tumor cells (CTCs) were enriched via negative selection by depleting CD45^+^ leukocytes using Magnetic‐Activated Cell Sorting (MACS). Anti‐CD45 magnetic microbeads (Miltenyi Biotec, Cat# 130‐045‐801) were used according to the manufacturer's protocol, and the CD45^−^ fraction was collected using LD columns (Miltenyi Biotec, Cat# 130‐042‐901) placed in a magnetic field.

Following enrichment, the CD45^−^ cell fraction was immediately prepared for immunocytochemistry. The cells were deposited onto poly‐L‐lysine‐coated slides using a cytocentrifuge (Shandon Cytospin) at 800 rpm for 5 min to ensure even distribution and optimal adherence. The slides were then air‐dried, fixed with 4% paraformaldehyde, and stored at −20°C until further staining and analysis.

### 2.4 CTC Phenotyping and Enumeration

To distinguish epithelial and mesenchymal subtypes of CTCs, immunofluorescent staining was performed using the following antibodies: EpCAM (rabbit anti‐human EpCAM, Padza, PDZMM113, 1:300) as an epithelial marker and secondary antibodies were Alexa Fluor 647‐conjugated donkey anti‐rabbit IgG (BioLegend, 406414). CD90 (mouse anti‐human CD90, BD Pharmingen, 550402, 1:1000) as a mesenchymal marker and FITC‐conjugated rat anti‐mouse IgG1 (BioLegend, 553443) were used as secondary antibodies. Nuclear staining was performed using DAPI to minimize the possibility of spectral bleed‐through; all images were acquired using channel‐specific excitation and emission filter sets. Exposure times were recorded across low‐to‐high settings during image acquisition to avoid missing rare CTC events. High‐exposure images were initially obtained to ensure sensitivity for detecting low‐abundance cells; however, images presented in the revised manuscript were reprocessed to reduce background fluorescence while preserving marker‐specific signals. Following fixation and staining, CD45^−^ cells were examined under a fluorescence microscope (Nikon Eclipse Ti2). CTCs were identified based on both immunophenotype (EpCAM^+^ and/or CD90^+^) and morphological criteria including cell size > 8 μm, high nuclear‐to‐cytoplasmic ratio, and irregular shape. These morphological characteristics were used only as qualitative supportive criteria during microscopy, and not as quantitatively measured endpoints, because the primary objective of the study was the enumeration of CTCs rather than detailed morphometric analysis. Fluorescence signals for EpCAM and CD90 were evaluated based on marker positivity rather than absolute fluorescence intensity, as the study was designed to classify and enumerate CTCs rather than quantify marker expression levels. To validate the MACS protocol and staining specificity, we used the human lung adenocarcinoma‐derived A549 cell line as a positive control. A549 cells naturally express epithelial markers such as EpCAM and are also capable of exhibiting mesenchymal traits under specific conditions, making them a suitable model for dual‐phenotype validation. Cells were cultured under standard conditions and subsequently spiked into PBMCs isolated from a healthy donor. These spiked samples underwent CD45‐negative magnetic separation and were subjected to the same staining and imaging procedures as clinical specimens, confirming the reliability and efficiency of the workflow.

Manual enumeration of CTCs was performed in all samples at both pre‐treatment and post‐gastrectomy time points and validated using ImageJ software.

#### 2.4.1 Risk Stratification Based on Perioperative CTC Dynamics

**FIGURE 2:**
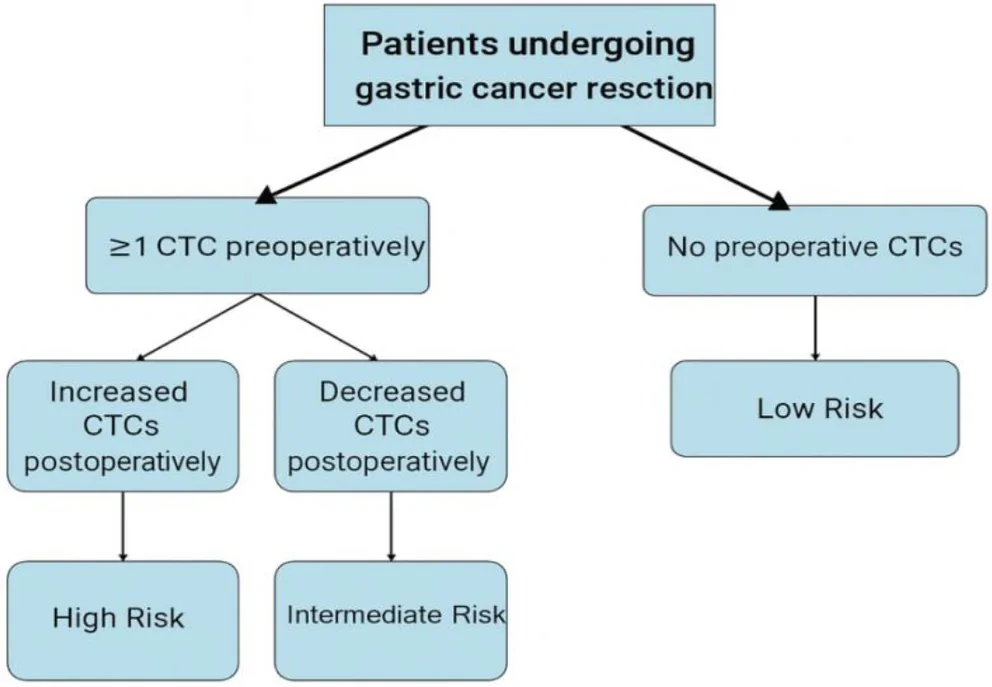
CTC‐based risk stratification algorithm for gastric cancer patients undergoing gastrectomy.

To investigate the prognostic implications of circulating tumor cell (CTC) kinetics in gastric cancer, patients were categorized into three distinct risk groups based on perioperative CTC enumeration (Figure 2):

- **High risk**: Patients with ≥ 1 CTC detected preoperatively and increased CTC counts postoperatively.
- **Intermediate risk**: Patients with ≥ 1 CTC detected preoperatively but decreased CTC counts following surgery.
- **Low risk**: Patients with no detectable CTCs either before or after surgery.

The CTC‐based risk classification into three groups was determined based on pre‐ and post‐operative CTC dynamics, informed by previously published studies and the distribution of counts in our cohort. Sensitivity analyses indicated that modest changes in cut‐off thresholds did not substantially alter the group assignments or study conclusions, supporting reproducibility.

### 2.5 Gene Expression Analysis in Gastric Tumor Tissue

A panel of ECM‐related genes—COL1A1, COL1A2, COL4A1, COL6A3, FN1, THBS2, and ITGB1—alongside miR‐29b was selected based on a prior in silico analysis of transcriptomic datasets related to extracellular matrix remodeling in gastric cancer. “The initial identification of these candidates was based on an unbiased transcriptomic and bioinformatics screening of publicly available gastric cancer datasets, and the present study was designed to experimentally validate these predefined ECM‐related genes rather than perform de novo high‐throughput gene discovery.”

This bioinformatics analysis, previously published, aimed to identify candidate genes with potential diagnostic or prognostic relevance [7]. Total RNA was extracted from frozen primary tumor tissues using AccuZol reagent, followed by DNase I treatment to eliminate genomic DNA. cDNA synthesis was performed using a combination of random hexamer and oligo(dT) primers. Quantitative real‐time PCR (qRT‐PCR) was conducted with SYBR Green Master Mix (Ampliqon). β‐actin was assessed as an internal control for mRNA targets, while U6 snRNA was evaluated for miRNA normalization. Primer specificity was confirmed by melt curve analysis and agarose gel electrophoresis. Primer sequences are listed in Table S1.

To explore potential clinical relevance, the relative expression levels of ECM‐related genes in tumor tissues were statistically analyzed in relation to clinicopathological parameters such as TNM stage, tumor grade, lymph node involvement, Metastasis, and family history. Statistical methods are detailed in the data analysis section. Based on combined bioinformatics ranking, expression consistency, and regulatory relevance, one candidate was prioritized for further expression analysis in circulating tumor cells (CTCs), aiming to assess its feasibility as a non‐invasive biomarker for gastric cancer progression.

### 2.6 Gene Expression Profiling in Circulating Tumor Cells

Peripheral blood samples collected prior to treatment were processed for CTC isolation using MACS‐based depletion of CD45^+^ leukocytes. The enriched CD45^−^fraction was used for total RNA extraction via TRIzol reagent (Invitrogen). RNA purity and quantity were assessed using a NanoDrop spectrophotometer. cDNA was synthesized using a non‐miRNA‐specific reverse transcription kit (FIREScript RT, Solis BioDyne), with optimized conditions to ensure efficient reverse transcription of small RNAs. Adjustments included increased RNA input, use of random hexamer primers, and controlled incubation temperature. Given the inherent limitations of MACS in fully eliminating leukocytes, potential background contamination was considered. Experimental conditions were designed to mitigate its effect on downstream gene expression analysis. qRT‐PCR was performed using SYBR Green (Ampliqon). U6 snRNA served as the internal reference gene.

In CTC‐negative patients and healthy control samples, miR‐29b expression was measured in the same CD45^−^ enriched fraction used for CTC isolation, even if no CTCs were detectable. This approach allows consistent comparison across all patient subgroups and controls, while minimizing potential background from leukocytes. For CTC‐positive patients, miR‐29b was measured specifically in the isolated CTC fraction. All samples were processed under identical conditions to ensure comparability of qRT‐PCR results.

### 2.7 Statistical Analysis

All statistical analyses were performed using SPSS v26 (IBM Corp.), GraphPad Prism v9, EZR (Jichi Medical University, Japan), and REST 2009 (Qiagen). A *p*‐value of < 0.05 was considered statistically significant. Patients were grouped as N0–N1 versus N2–N3 to reflect limited versus extensive nodal involvement. A supplementary analysis using N0 versus *N*+ was performed to validate robustness.

Continuous normally distributed data were analyzed using *t*‐tests or ANOVA, while non‐parametric data were analyzed with Wilcoxon signed‐rank tests. Categorical variables were compared using Chi‐square or Fisher's exact tests as appropriate. Kaplan–Meier survival analysis with log‐rank test was applied to evaluate relapse‐free survival. Correlations between gene expression and clinical parameters were assessed with Pearson or Spearman coefficients, depending on data distribution.

#### 2.7.1 CTC Enumeration and Phenotyping

The association between CTC presence or phenotype and clinicopathological variables was assessed using Fisher's exact test or Chi‐square test, as appropriate. Kaplan–Meier survival curves were used to evaluate relapse‐free survival (RFS), with differences compared using the log‐rank test.

#### 2.7.2 Gene Expression Analyses

Relative expression levels of target genes were calculated using the 2^−ΔΔCt^ method. For tissue‐based comparisons, paired or unpaired *t*‐tests and ANOVA were used. For CTC‐based expression, pooled RNA from healthy donors served as the reference. “For comparisons of miR‐29b expression across multiple clinical subgroups, one‐way analysis of variance (ANOVA) was used to assess overall group differences, followed by Tukey's post hoc test where applicable. This approach was applied to reduce multiple pairwise comparisons and strengthen statistical inference.”

All reactions were normalized against appropriate internal controls. Data were evaluated using REST software and visualized with GraphPad Prism.

#### 2.7.3 Correlation and Diagnostic Evaluation

Pearson or Spearman correlation coefficients were used to assess associations between gene expression and clinical parameters. Receiver operating characteristic (ROC) curve analyses were performed to determine the diagnostic performance of expression signatures.

## 3 Result

### 3.1 Results From Preliminary Studies Informing Experimental Design

To guide the experimental design, two prior investigations were conducted:

1. Systematic review and meta‐analysis

A comprehensive synthesis of published data on circulating tumor cells (CTCs) in gastric cancer highlighted the clinical relevance of CTC detection. Among the various enrichment strategies, Magnetic‐Activated Cell Sorting (MACS) emerged as a feasible and reproducible approach. Furthermore, studies incorporating immunofluorescent phenotyping, particularly using epithelial (EpCAM) and mesenchymal (CD90) markers, showed higher sensitivity in detecting phenotypic diversity of CTCs and their potential association with disease progression.

2 Bioinformatics analysis of ECM‐related genes and regulatory miRNAs

A transcriptomic analysis of publicly available gastric cancer datasets was conducted to identify extracellular matrix (ECM)‐related genes and associated regulatory microRNAs involved in tumor progression and metastasis. Pathway enrichment analysis revealed several ECM remodeling genes, including COL1A1, COL1A2, COL4A1, COL6A3, FN1, THBS2, and ITGB1, to be consistently dysregulated. Among predicted regulatory microRNAs, miR‐29b was identified as a key modulator of ECM components and was downregulated in gastric cancer tissues.

These findings provided the rationale for:

- Selecting MACS and dual‐marker immunofluorescence as the primary CTC detection method.
- Prioritizing miR‐29b and ECM‐related genes for experimental validation in both tumor tissue and CTCs.

### 3.2 Patient Demographics and Clinical Characteristics

A total of 30 patients with histologically confirmed gastric adenocarcinoma were enrolled in the study. The mean age was 63.1 years (SD ±8.5), with a range of 43 to 83 years. The cohort comprised 66.7% male (*n* = 20) and 33.3% female (*n* = 10) participants.

Tumor staging at diagnosis revealed that the majority of patients were in advanced stages: 60% (*n* = 18) were Stage III, followed by 16.7% in Stage II, 13.3% in Stage IV, and 10% in Stage I. Lymph node involvement was observed across various levels: 33.3% (N0), 37.7% (N1), 10.1% (N2), and 18.9% (N3). Regarding the depth of tumor invasion, T2 lesions were the most prevalent (46.4%), followed by T3 (26.1%), T1 (17.4%), and T4 (10.1%).

At the time of enrollment, distant metastasis was present in 33.3% of patients (*n* = 10), while the remaining 66.7% (*n* = 20) were non‐metastatic. These baseline characteristics provide the clinical context for evaluating circulating tumor cell (CTC) presence and ECM‐related gene expression in subsequent analyses (Table 1).

**TABLE 1:** Baseline demographic and clinicopathological characteristics of patients with gastric cancer.

Parameter	Value
Age (years, mean ± SD)	61.8 ± 11.7
Age range (years)	32–83
Sex—Male	20 (66.7%)
Sex—Female	10 (33.3%)
Stage I	3 (10%)
Stage II	5 (16.7%)
Stage III	18 (60%)
Stage IV	4 (13.3%)
T1	12 (17.4%)
T2	32 (46.4%)
T3	18 (26.1%)
T4	7 (10.1%)
N0	23 (33.3%)
N1	26 (37.7%)
N2	7 (10.1%)
N3	13 (18.8%)
Metastatic	10 (33.3%)
Non‐metastatic	20 (66.7%)

### 3.3 Phenotypic Characterization of Circulating Tumor Cells (CTCs) Before and After Treatment

**FIGURE 3:**
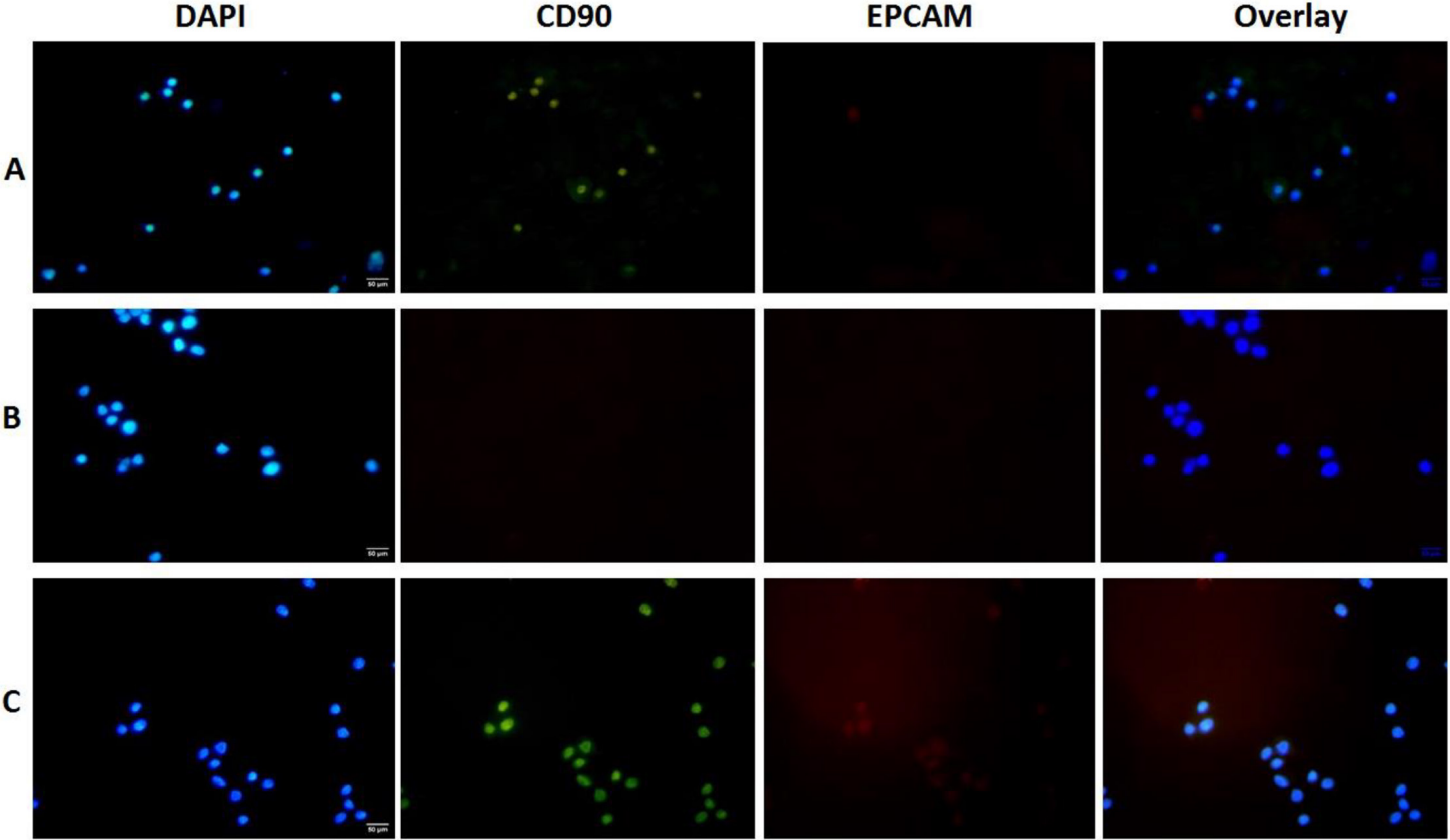
Fluorescence labeling of circulating tumor cells (CTCs) for DAPI, CD90, and EPCAM markers. (A) CTCs from a patient with gastric cancer showing positive staining for the mesenchymal marker CD90 (green) and nuclear DAPI (blue), but negative for the epithelial marker EPCAM (red). This phenotype reflects a mesenchymal CTC profile. (B) Negative control using peripheral blood from a healthy donor. Only DAPI‐positive nuclei are visible, with no detectable signal for CD90 or EPCAM, confirming staining specificity. (C) Positive control using A549 lung carcinoma cells. Cells exhibit strong dual expression of CD90 (green) and EPCAM (red), with DAPI (blue) marking the nuclei, validating antibody performance. Images were acquired using channel‐specific excitation and emission filters. Representative images were reprocessed to reduce background fluorescence. Magnification: 40×, Scale bar = 20 μm. Negative‐control samples from healthy donors showed no detectable CD90 or EPCAM fluorescence even under high‐exposure conditions, confirming the absence of channel bleed‐through. In contrast, A549 positive control cells demonstrated clear and distinct CD90 and EPCAM signals, validating antibody specificity. Representative images in the revised figure have been reprocessed to reduce background fluorescence and enhance signal‐to‐noise ratio.

To assess the phenotypic dynamics of circulating tumor cells (CTCs) in response to treatment, immunofluorescence‐based phenotyping was conducted at two time points: prior to initiation of therapy and approximately 1 month after completion of gastrectomy combined with chemotherapy. CTCs were classified into two major phenotypes based on surface marker expression: epithelial (EpCAM^+^) and mesenchymal (CD90^+^) (Figure 3A).

To confirm the reliability of the MACS‐based enrichment strategy for detecting both epithelial and mesenchymal CTC phenotypes, the A549 lung adenocarcinoma cell line was used as a positive control. Following spiking of A549 cells into PBMCs from a healthy donor and CD45‐negative selection, immunofluorescent staining successfully identified both EpCAM^+^ and CD90^+^ populations, validating the capability of the method to recover heterogeneous CTC subtypes. Optimal antibody dilutions for EpCAM and CD90 were determined by testing 1:100 and 1:300 concentrations on murine spleen cells. Both showed clear signal, and 1:300 was selected for further phenotypic analysis due to adequate intensity and specificity. The observed fluorescence signals were restricted to their corresponding channels, and no cross‐channel signal contamination was detected during the imaging workflow. This was further confirmed by the complete absence of CD90 and EpCAM signals in negative‐control samples, supporting the specificity of fluorescence detection (Figure 3B,C).

Morphological characteristics were used as supportive qualitative criteria during CTC identification; however, detailed morphometric measurements such as exact cell diameter, nuclear‐to‐cytoplasmic ratio, or shape irregularity were not quantified separately, as the study endpoints focused on CTC enumeration. At baseline, mesenchymal CTCs were the predominant subtype, with a mean count of 14.07 cells per 10 mL of peripheral blood (range: 0–98). In contrast, epithelial CTCs were infrequently detected, with a baseline mean of 0.5 cells (range: 0–3). Following treatment, mesenchymal CTCs were significantly reduced (mean: 1.18; *p* = 0.0064, paired *t*‐test; *p* = 0.0002, Wilcoxon signed‐rank test), and epithelial CTCs also showed a statistically significant decrease (mean: 0.09; *p* = 0.0246, paired *t*‐test; *p* = 0.0258, Wilcoxon test), although their absolute levels remained low. Among the 22 patients with paired measurements, 15 exhibited complete clearance of mesenchymal CTCs and 20 had no detectable epithelial CTCs after therapy. A detailed summary of the statistical test results is presented in Table S2.

### 3.4 Enumeration and Comparative Analysis of Circulating Tumor Cells (CTCs) Among Clinicopathological Subgroups

To assess the relationship between circulating tumor cell (CTC) presence and clinicopathological characteristics, CTC enumeration—including total CTCs, epithelial (EpCAM+) phenotype, and mesenchymal (CD90+) phenotype—was compared across key clinical subgroups: metastasis status (M0 vs. M1), lymph node involvement (N0–1 vs. N2–3), tumor stage (Stage I–II vs. III–IV), and tumor invasion depth (T1–2 vs. T3–4). As shown in Table 2, no statistically significant associations were found between CTC positivity and any of the clinical variables (all *p*‐values > 0.05, Fisher's exact test). While higher proportions of CTC+ patients were observed in earlier stage and node‐negative groups, these differences did not reach statistical significance. These results indicate that, within this cohort, the detection of total or phenotype‐specific CTCs was not significantly influenced by tumor stage, lymph node status, metastasis, or depth of invasion.

**TABLE 2:** Distribution and statistical comparison of CTC positivity across clinical subgroups.

Clinical variable	CTC+ (%)	CTC epithelial+ (%)	CTC mesenchymal+ (%)	*p* (CTC)	*p* (epithelial)	*p* (mesenchymal)
Metastasis (M0 vs. M1)	33.3 versus 33.3	16.7 versus 8.3	33.3 versus 33.3	1	0.632	1
Node (N0‐1 vs. N2‐3)	46.2 versus 23.5	15.4 versus 11.8	46.2 versus 23.5	0.255	1	0.255
Stage (I‐II vs. III‐IV)	50 versus 18.8	14.3 versus 12.5	50 versus 18.8	0.122	1	0.122
Tumor (T1‐2 vs. T3‐4)	50 versus 22.2	16.7 versus 11.1	50 versus 22.2	0.139	1	0.139

*Note:* The values represent the proportion of patients with detectable total CTCs, epithelial phenotype (EpCAM+), or mesenchymal phenotype (CD90+). All *p*‐values are derived from Fisher's exact test.

### 3.5 Risk Stratification Based on Perioperative CTC Dynamics

In the final risk classification, a total of 25 patients were included. This count incorporates three deceased patients who were retrospectively assigned to risk categories based on available clinical and laboratory data. Accordingly, 17 patients (68%) were classified as intermediate risk, 4 patients (16%) as low risk, and 4 patients (16%) as high risk. This distribution indicates that the majority of patients demonstrated a reduction in CTCs following therapy, thereby falling into the intermediate‐risk category.

### 3.6 Kaplan–Meier Survival Curves and Statistical Comparison

**FIGURE 4:**
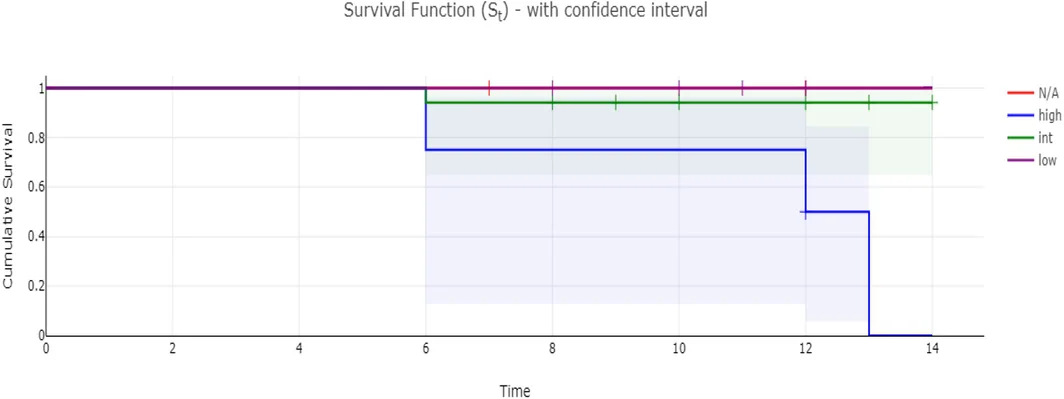
Kaplan–Meier survival curves for high‐, intermediate‐, risk patients. Shaded regions represent confidence intervals. Due to the limited follow‐up duration, survival curves should be interpreted as indicative of biological risk stratification rather than long‐term outcome prediction.

To further evaluate the prognostic value of CTC‐based risk stratification, Kaplan–Meier survival curves were generated for all three risk groups (Figure 4). Patients were categorized into Low, Intermediate, and High Risk groups based on CTC dynamics before and after treatment.

The High Risk group demonstrated a significant decline in survival probability, with all observed death events occurring within this group during the 14‐month follow‐up. In contrast, the Intermediate Risk group exhibited stable survival with minimal decline, while the Low Risk group maintained a 100% survival rate throughout the observation period.

The survival curves display a clear separation between the High Risk group and the other two categories, underscoring the prognostic relevance of elevated postoperative CTC levels. The absence of death events in the Low Risk group and prolonged survival in the Intermediate group further supports the clinical utility of CTC‐based stratification for risk assessment and individualized patient monitoring following gastrectomy in gastric cancer.

To assess survival differences between CTC‐based risk groups, a log‐rank test was performed. The analysis yielded a chi‐square statistic of 11.16 with 2 degrees of freedom, and the corresponding *p*‐value was approximately 0.0107, indicating a statistically significant difference in survival distributions across the groups. The Phi effect size (Φ) was calculated at 1.67, suggesting a strong association between CTC‐based risk stratification and survival outcome. Most death events occurred in the high‐risk group, while no deaths were observed in the low‐risk group. These findings align with Kaplan–Meier curves and support the prognostic significance of postoperative CTC dynamics in gastric cancer.

### 3.7 Gene Expression Results in Tumor Tissue and Circulating Tumor Cells (CTCs)

To validate the bioinformatics predictions regarding the involvement of extracellular matrix (ECM) remodeling genes in gastric cancer, we evaluated the expression levels of selected ECM‐related genes in tumor tissues of gastric cancer patients compared to normal tissues. Among the assessed genes, THBS2, FN1, COL6A3, COL4A1, COL1A2, and miR‐29b were significantly downregulated in tumor tissues, while COL1A1 showed significant upregulation (fold change = 2.05, *p* = 0.01158). No significant expression change was observed for ITGB1 (*p* = 0.93103). Given the consistency between bioinformatics predictions and experimental tissue‐level expression for miR‐29b, and its recognized role in regulating ECM and tumor suppression, miR‐29b was selected for further validation in CTCs. Subsequent analysis revealed that miR‐29b was significantly downregulated in CTCs isolated from gastric cancer patients (pre‐chemotherapy and post‐gastrectomy) compared to healthy controls (fold change = 0.2049, *p* < 0.0001), indicating its potential utility as a non‐invasive biomarker for gastric cancer. These data are summarized in Table 3.

**TABLE 3:** Gene expression analysis of ECM pathway‐related genes in tumor tissue and CTC compared to normal control.

Gene	Status	Fold change	Regulation	*p*
THBS2	Tumor versus normal	0.0385	Downregulated	< 0.0001
FN1		0.2067	Downregulated	< 0.0001
ITGB1		1.028	No change	0.9310
COL6A3		0.1293	Downregulated	< 0.0001
COL1A1		2.0548	Upregulated	0.0116
COL4A1		0.062	Downregulated	< 0.0001
COL1A2		0.0126	Downregulated	< 0.0001
miR‐29b		0.5163	Downregulated	0.0136
miR‐29b	CTC versus normal	0.2049	Downregulated	< 0.0001

### 3.8 Association of Gene Expression of miR‐29b in CTCs With Clinical Demographic Variables

To evaluate the potential clinical significance of miR‐29b expression in circulating tumor cells (CTCs), –ΔCt values were analyzed across clinicopathological variables, including tumor stage, tumor depth (T category), nodal status, metastatic status, CTC detection status, family history, and CTC‐based risk groups. Overall group differences were first assessed using one‐way analysis of variance (ANOVA) to reduce multiple pairwise comparisons.

Within the patient cohort, no statistically significant differences in miR‐29b expression were detected across tumor stage (ANOVA *p* = 0.667), tumor depth (*p* = 0.667), nodal status (*p* = 0.747), metastatic status (*p* = 0.248), CTC detection status (*p* = 0.575), family history (*p* = 0.381), or CTC‐dynamics risk groups (*p* = 0.826) (Table 4). These findings indicate that, although miR‐29b is broadly downregulated in gastric cancer, its expression within circulating tumor cells does not significantly differ among clinicopathological subcategories of affected patients.

**TABLE 4:** Differential expression of miR‐29b in CTCs across clinical subgroups.

Clinical variable	Subgroups	ANOVA *p* (overall)
Tumor stage	Stage I–II versus Stage III–IV	0.667
Tumor depth (T)	T1–2 versus T3–4	0.667
Node status (N)	N0–1 versus N2–3	0.747
Metastasis (M)	M0 versus M1	0.248
CTC detection status	CTC+ versus CTC−	0.575
Family history	Yes versus no	0.381
CTC‐based risk groups	Low versus intermediate versus high	0.826

*Note:* Overall group differences in miR‐29b expression (–ΔCt) were assessed using one‐way analysis of variance (ANOVA). For comparisons involving more than two groups, Tukey's post hoc test was applied where appropriate. Statistical significance was defined as *p* < 0.05.

When comparing all tumor cases collectively against normal controls, miR‐29b expression was significantly reduced in the tumor group (*p* = 0.0136), supporting its overall diagnostic relevance in gastric cancer.

### 3.9 Diagnostic Potential of miR‐29b Expression in Gastric Cancer

**FIGURE 5:**
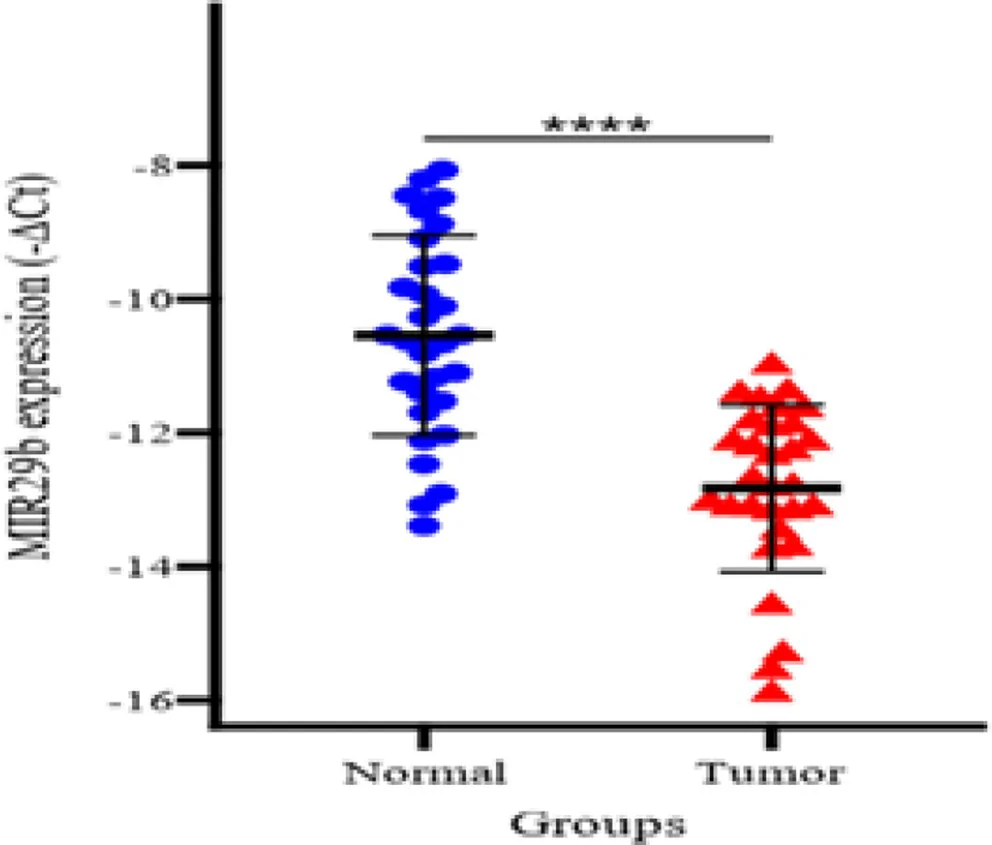
Expression levels of miR‐29b (–ΔCt) in normal versus tumor gastric tissues. The plot illustrates the magnitude of differential expression between groups.

To investigate the diagnostic relevance of miR‐29b, its expression was assessed in paired gastric tumor and adjacent normal tissues using quantitative real‐time PCR (qPCR). As shown in Figure 5, tumor samples exhibited significantly lower –ΔCt values compared to normal tissues, indicating reduced miR‐29b expression in gastric cancer (*p* = 0.0136). This trend was consistent in both dot and box plot visualizations, supporting the robustness of the observation.

To further evaluate its discriminatory capacity, ROC curve analysis was conducted. The area under the ROC curve (AUC) was 0.883 (95% CI: 0.798–0.969), demonstrating strong diagnostic performance. The optimal threshold (ΔCt = 11.314) yielded a sensitivity of 96.7% and a specificity of 70%, suggesting that miR‐29b could serve as a reliable biomarker for distinguishing gastric tumor tissue from normal mucosa.

## 4 Discussion

In this study, we integrated bioinformatics and experimental platforms to explore the role of circulating tumor cells (CTCs) and miR‐29b expression in gastric cancer. Utilizing a MACS‐based enrichment strategy combined with epithelial/mesenchymal immunophenotyping and qPCR, we effectively detected heterogeneous CTC populations, including in advanced‐stage patients. miR‐29b was significantly downregulated in both tumor tissues and CTCs, supporting its diagnostic relevance. The CD45‐negative MACS protocol proved technically robust, enabling reliable CTC isolation, with particularly high counts in some stage III/IV cases—suggesting an aggressive phenotype. Validation with A549 cell lines confirmed the system's specificity. These findings are consistent with prior studies, including Ishiguro et al. [6], underscoring the prognostic potential of mesenchymal CTC profiling in gastric cancer.

The primary advantage of our MACS protocol lies in its simplicity, reproducibility, and compatibility with downstream molecular analyses. Unlike label‐dependent or microfluidic systems, MACS efficiently captures heterogeneous CTC populations, including those undergoing epithelial‐to‐mesenchymal transition (EMT)—a key limitation of EpCAM‐based methods. Given the phenotypic plasticity of CTCs during metastasis, this broader capture is critical. Prior studies, such as Toyoshima et al. [8], have demonstrated that CTCs from gastric cancer patients possess tumorigenic potential in vivo, underscoring their clinical relevance. CTC imaging provides morphological context, enabling identification of viable cells, which we aim to evaluate in head‐to‐head comparison studies with circulating tumor DNA (ctDNA) assays to determine the complementary prognostic value of cellular versus cell‐free biomarkers in relapse risk stratification. Our previous meta‐analysis further supports the utility of non‐EpCAM‐based methods, showing that the prognostic value of CTCs persists across detection platforms and phenotypes, affirming the broader clinical applicability of technologies like MACS [4]. The consistency between positive‐control staining, the complete absence of marker signals in negative controls, and the lack of channel bleed‐through collectively support the reliability of our immunofluorescence‐based CTC detection strategy. Although detailed morphometric quantification was beyond the scope of the study, qualitative morphological assessment supported marker‐based CTC identification.

In our cohort, mesenchymal CTCs (CD90^+^) were markedly more prevalent than epithelial CTCs (EpCAM^+^), with mean baseline counts of 14.7 and 0.5 cells per 10 mL of blood, respectively. Post‐treatment analyses revealed a statistically significant reduction in mesenchymal CTCs (*p* = 0.0064), while epithelial CTCs, already infrequent, further declined (*p* = 0.0246). Both paired *t*‐tests and Wilcoxon signed‐rank tests confirmed these trends. To directly investigate this clinical correlation, we plan to longitudinally track CD90^+^ CTC dynamics in patients undergoing active chemotherapy to establish whether persistence of this subpopulation serves as a functional indicator of therapeutic resistance. These results underscore the need to include mesenchymal markers such as CD90 in CTC detection protocols to avoid underestimating the true CTC burden, particularly in advanced gastric cancer.

Notably, mesenchymal CTCs (CD90^+^) were detected in 25 of 30 patients (83.3%) at baseline—exceeding the prevalence reported in previous studies. Wu et al. [9] identified mesenchymal markers in 53% of cases, while Nel et al. [10] and Ishiguro et al. [6] observed N‐cadherin^+^ CTCs in 35% of gastric cancer patients, linking their presence to poor prognosis and recurrence. The higher detection rate in our study may reflect the broader sensitivity of CD90 in capturing EMT‐associated CTCs. Based on these findings, our group plans to utilize CD90‐based CTC enrichment in our upcoming prospective trials to refine patient stratification and monitor early systemic dissemination in high‐risk gastric cancer phenotypes. While the predominance of mesenchymal CTCs in our cohort highlights the phenotypic plasticity of circulating tumor cells in gastric cancer, a notable observation was the persistence of CD90^+^ CTCs in a subset of patients despite curative‐intent surgery and chemotherapy. This residual presence may reflect early micrometastatic dissemination or resistance to standard treatments. Notably, no significant association was found between CTC status and conventional clinicopathological parameters—including tumor stage, nodal involvement, or distant metastasis—suggesting that CTC dynamics may be influenced by biological factors beyond traditional anatomical staging. Importantly, CD90 should not be considered a replacement for established epithelial markers, but rather a complementary marker that enables detection of mesenchymal CTC sThe persistence of mesenchymal CTCsubpopulations undergoing epithelial–mesenchymal transition, which are frequently underrepresented in EpCAM‐based platforms. Although CTC detection did not significantly differ across nodal groups, the use of a biologically meaningful N0–N1 versus N2–N3 classification allowed us to explore whether CTC phenotypes reflect tumor burden and lymphatic dissemination patterns.

To date, only two studies—including the present work and that by Ishiguro et al. [6]—have established a perioperative CTC‐based risk stratification model in gastric cancer. Both adopted a shared classification system, grouping patients into high, intermediate, and low‐risk categories according to CTC status before and after surgery, enabling meaningful comparative analysis. While the Ishiguro study reported a predominance of low‐risk patients (65%), our cohort was largely composed of intermediate‐risk cases (68%), with only 16% falling into the low‐risk group. This difference may reflect methodological variations, particularly the use of CD90 in our protocol, which may enhance detection of mesenchymal CTCs and increase baseline positivity rates. Despite these differences, both studies consistently demonstrated that high‐risk patients experienced the poorest outcomes, reinforcing the prognostic value of CTC‐based risk stratification in gastric cancer. However, the survival analysis presented here is intended to illustrate the prognostic relevance of perioperative CTC dynamics for risk stratification rather than to provide definitive long‐term survival estimates, particularly given the relatively short follow‐up period.

A key focus of our study was the expression profile of miR‐29b in CTCs. We found significant downregulation of miR‐29b in both tumor tissues and CTCs from gastric cancer patients compared to healthy controls, supporting its role as a tumor‐associated molecular alteration. However, within the patient cohort, miR‐29b expression did not significantly differ across clinicopathological subgroups or CTC‐based risk categories, suggesting that its reduction may reflect a global feature of gastric carcinogenesis rather than a stage‐ or risk‐specific discriminator. These results suggest a tumor‐suppressive role for miR‐29b, consistent with its regulatory influence on extracellular matrix remodeling, EMT, and metastasis. Our bioinformatic analysis [7] initially highlighted several ECM‐related genes—such as THBS2, FN1, COL1A1, ITGB1, and COL6A3—alongside miR‐29b. However, experimental validation confirmed consistent expression patterns only for miR‐29b and COL1A1, underscoring the importance of integrating in silico predictions with empirical data. This divergence may reflect complex post‐transcriptional mechanisms or tumor microenvironmental factors not captured in computational models. Accordingly, Table 3 reflects an experimentally validated subset of ECM‐related candidates derived from prior unbiased bioinformatics analyses, rather than the results of a new discovery‐based screening. miR‐29b is increasingly recognized as a key regulator of tumorigenesis in diverse malignancies. Yan et al. [11, 12] demonstrated that miR‐29b modulates epigenetic remodeling by directly targeting DNA methylation‐related enzymes, including DNMTs, TET1, and TDG, thereby altering the epigenetic landscape of cancer cells. Additionally, miR‐29b has been implicated in promoting apoptosis via downregulation of anti‐apoptotic proteins such as Mcl‐1 and BCL2, as well as activation of the tumor suppressor p53 [13]. These findings underscore its multifaceted tumor‐suppressive functions.

In gastric cancer, our findings corroborate previous reports identifying miR‐29b as a suppressor of EMT and metastatic progression [14]. Its consistent downregulation in gastric cancer patients supports its potential diagnostic relevance, although its independent prognostic utility requires further validation. Mechanistically, miR‐29b may exert anti‐tumor effects through inhibition of ECM remodeling and repression of pro‐invasive signaling pathways such as TGF‐β and NF‐κB, both of which are known to suppress miR‐29b expression [15]. Preclinical studies further demonstrate that synthetic miR‐29b mimics can induce apoptosis, reduce tumor burden, and alter the cancer methylome [16], highlighting its therapeutic promise. While challenges remain regarding miRNA delivery and specificity, our data add to the growing evidence establishing miR‐29b as a central regulator in gastric cancer pathobiology [17]. While miR‐29b has been extensively implicated in gastric cancer biology, its expression has not previously been specifically evaluated in circulating tumor cells of gastric cancer patients. Our results revealed significantly reduced levels of miR‐29b in all patient subgroups compared to healthy controls. Interestingly, no significant differences were observed between tumor stages or metastatic status, suggesting that miR‐29b downregulation appears independent of classical clinicopathological parameters. These findings align with previous studies reporting the tumor‐suppressive role of miR‐29b via targeting of epigenetic regulators [18], apoptosis‐related genes [19], and EMT‐associated pathways [20]. The association of miR‐29b suppression with CTC presence highlights its potential utility as both a non‐invasive biomarker and a therapeutic target in aggressive gastric cancer phenotypes. This study represents the first evaluation of miR‐29b expression in circulating tumor cells of gastric cancer patients in the context of perioperative CTC dynamics. Although miR‐29b expression was consistently reduced in cancer patients compared to healthy controls, no statistically significant differences were observed among CTC‐based risk groups. These findings suggest that miR‐29b downregulation may represent a fundamental molecular alteration in gastric cancer rather than a marker capable of independently distinguishing clinical risk categories. Additionally, miR‐29b expression was significantly reduced in gastric tumor tissues compared to adjacent normal mucosa. ROC analysis confirmed the diagnostic potential of miR‐29b, with an AUC of 0.883, high sensitivity (96.7%), and acceptable specificity (70%). Together, these findings reinforce the dual diagnostic and prognostic value of miR‐29b in gastric cancer and underscore the clinical relevance of integrating miRNA profiling with CTC‐based risk models.

From a clinical standpoint, we plan to utilize the detection of CD90‐positive mesenchymal CTCs going forward to monitor longitudinal therapeutic response, evaluate its ability to identify patients who will benefit from aggressive adjuvant therapies, and guide more intensive post‐operative surveillance protocols within our cohort.

In addition, while CTC‐based assays are not currently recommended for population‐wide screening, their potential utility as a complementary monitoring tool in high‐risk individuals—such as those with premalignant gastric lesions or a strong family history—warrants further investigation. Prospective multicenter studies will be necessary to determine whether CD90‐enriched CTC detection can be incorporated into early detection or surveillance algorithms in gastric cancer.

This study presents an integrated molecular and clinical analysis of CTC dynamics in gastric cancer but is subject to several limitations. One limitation of this study is the relatively small sample size, which reflects the technical and logistical challenges of collecting paired perioperative circulating tumor cell samples from gastric cancer patients. Accordingly, the present work should be regarded as an exploratory, hypothesis‐generating study, and future multicenter investigations with larger cohorts are required to validate these findings.

Biological heterogeneity among patients, including genetic and epigenetic variability, may have influenced CTC and gene expression profiles. While MACS enabled enrichment of both epithelial and mesenchymal CTCs, technical constraints such as marker dependency, cell loss, and inability to isolate hybrid EMT phenotypes limit its sensitivity. Additionally, discrepancies between bioinformatic predictions and qPCR validation highlight challenges in translational interpretation. Lastly, limited long‐term follow‐up data precluded robust assessment of miR‐29b's prognostic value over time. Future multicenter studies with extended follow‐up and functional validation are warranted to substantiate these findings.

Although CD90 demonstrated strong sensitivity for detecting mesenchymal CTCs, our study was not designed to perform a comprehensive comparison with other established mesenchymal markers (e.g., CD44, N‐cadherin, vimentin). Such comparative analyses, as well as multivariate models incorporating CD90 status alongside clinicopathological factors, require substantially larger cohorts to ensure adequate statistical power. Therefore, the present findings should be considered hypothesis‐generating, and future multicenter studies with expanded marker panels are warranted.

The relatively short follow‐up period (14 months) and the limited number of survival events—all of which occurred in the high‐risk group—restrict the robustness of our survival analyses. The low event count prevented more comprehensive statistical approaches such as multivariate Cox regression and may introduce bias in interpreting early mortality within this subgroup. Larger prospective cohorts with extended follow‐up are required to validate these survival trends and strengthen risk‐stratification based on perioperative CTC dynamics.

Building on the findings of this study, several directions for future research are recommended. First, longitudinal cohort studies with extended follow‐up are needed to validate the prognostic utility of miR‐29b expression and CTC enumeration in predicting survival and recurrence. Second, expanding the sample size and conducting multicenter trials will enhance statistical power and generalizability of the findings, particularly regarding the role of CD90^+^ mesenchymal CTCs. Third, advancements in CTC isolation methods—such as combining MACS with microfluidic or flow cytometry‐based platforms—may improve detection sensitivity and reduce cell loss during manual handling. Additionally, functional studies of miR‐29b in gastric cancer models are essential to elucidate its mechanistic role in invasion and metastasis. Finally, investigating related microRNAs (e.g., miR‐29a/c) and their involvement in ECM remodeling pathways such as TGF‐β and PI3K‐AKT signaling could provide a broader understanding of tumor progression and identify novel therapeutic targets.

Looking ahead, our group is actively pursuing the validation of these findings in a larger prospective cohort. We are performing deeper molecular characterization of CD90^+^ CTCs, including the evaluation of co‐expression with immune checkpoint markers such as PD‐L1 and stemness markers like nestin. Furthermore, we have initiated genomic profiling of these isolated CTCs to identify signatures of therapeutic resistance. Unlike ctDNA profiling, which reflects bulk circulating tumor burden, isolating viable CTCs allows us to link molecular markers like miR‐29b to specific phenotypic subpopulations, such as CD90^+^ mesenchymal cells, thereby providing critical cellular context. Consequently, we are designing studies to perform head‐to‐head comparisons of CD90^+^ CTC dynamics with ctDNA to establish an integrated liquid biopsy platform for gastric cancer management.

## 5 Conclusion

In summary, this study supports the combined diagnostic and prognostic relevance of perioperative CTC dynamics and miR‐29b expression in gastric cancer. Dual‐marker MACS‐based enrichment targeting both epithelial (EpCAM) and mesenchymal (CD90) phenotypes improved CTC detection sensitivity and enabled broader identification of potentially invasive cell populations. Risk stratification based on perioperative CTC changes was clinically informative, as elevated postoperative CTC burden was associated with poorer survival outcomes. In addition, miR‐29b was consistently downregulated in tumor tissues, peripheral blood, and isolated CTCs, supporting its potential value as a non‐invasive biomarker of disease progression. Given the exploratory nature and limited sample size of the present study, our next step will be to validate these findings in a larger prospective cohort and to further characterize CD90^+^ CTCs through expanded molecular profiling in order to better define their biological and clinical significance in gastric cancer.

## Author Contributions

M.R.E. conceptualized and designed the study, performed experiments, and drafted the manuscript. S.E. contributed to data analysis, interpretation, and manuscript editing. A.S.F. assisted in supervising the research process and contributed to the critical revision and editing of the manuscript. K.R. provided oncological supervision and oversaw the chemotherapy protocols. H.M., as a surgical oncology expert, defined the surgical protocol and guided postoperative management. M.T., as a gastroenterologist, was responsible for patient diagnosis and referral to oncology. F.G.M. supported the laboratory experiments and assisted M.R.E. in molecular procedures. M.A.O., as a medical physicist, helped optimize the MACS technique and experimental setup. R.S. provided senior supervision and final critical revision of the manuscript. All authors reviewed and approved the final manuscript.

## Conflicts of Interest

The authors declare no conflicts of interest.

## Supporting information


**Table S1:** Primer sequences from ECM‐related genes—COL1A1, COL1A2, COL4A1, COL6A3, FN1, THBS2, ITGB1and miR‐29b.

**Table S2:** Statistical test results for CTC changes before and after treatment.

**Table S3:** Differential expression of miR‐29b across CTC risk groups.

**Table S4:** Statistical test results for CTC changes before and after treatment.

## Data Availability

The data that support the findings of this study are available within the article and its Supporting Information.
