# Supplementary material for: Integrated Molecular and Phenotypic Profiling of Circulating Tumor Cells in Gastric Cancer: A Translational Perspective
Source: FASEB Bioadv. 2026 Aug 19;8(8):e70140. doi: 10.1096/fba.2025-00314 (PMC13489073; doi:10.1096/fba.2025-00314)
Supplement: Supplementary file 2 [file FBA2-8-e70140-s002.docx]

Supplementary Table 1. Primer sequences from ECM-related genes—COL1A1, COL1A2, COL4A1, COL6A3, FN1, THBS2, ITGB1and miR-29b.

| **Name** | **Type** | **Sequence (3' → 5')** | **nucleotide** |
| --- | --- | --- | --- |
|  |  |  |  |
| FITGB1 | mRNA | TGCCTTGGTGTCTGTGCTGAG | 21 |
| RITGB1 |  | TGGGACACAGGATCAGGTTGGA | 22 |
| FCOL1A1 | mRNA | CCCCTGGTGCTACTGGTTTCCC | 22 |
| RCOL1A1 |  | GACCTTTGCCGCCTTCTTTGC | 21 |
| FCOL4A1 | mRNA | TCGCTGTGGATCGGCTACTCT | 21 |
| RCOL4A1 |  | ACCATTCAGATCCCACCGTGC | 21 |
| FCOL1A2 | mRNA | CCTGGTGCCCCTGGTGAAAA | 20 |
| RCOL1A2 |  | ACTTCCATCACTGCCACGGG | 20 |
| FCOL6A3 | mRNA | ACACGCCTTCAGGTTTGCTCA | 21 |
| RCOL6A3 |  | GCAGCCGCACCATTTTTGACAT | 22 |
| FFN1 | mRNA | CCAAGACCATACCCGCCGAAT | 21 |
| RFN1 |  | AGGCATTTGGATTGAGTCCCG | 21 |
| FTHBS2 | mRNA | GAATTGCAAAGGGAGTGGCCG | 21 |
| RTHBS2 |  | AGGCTCAGGGCTGTTGCAGA | 20 |

| **Name** | **Type** | **Sequence (3' → 5')** | **nucleotide** |
| --- | --- | --- | --- |
| RT primer miR-29b-3p | miRNA | GTCGTATCCAGTGCAGGGTCCGAGGTATTCGCACTGGATACGACAACACT | 50 |
| miR-29b-3p (Forward) | miRNA | GTAGCACCATTTGAAATCAGTGTT | 24 |
| miR-29b-3p/U6 (Reverse) | miRNA | CAGTGCAGGGTCCGAGGT | 18 |
| RT primer U6 | miRNA | GTCGTATCCAGTGCAGGGTCCGAGGTATTCGCACTGGATACGACCGAATT | 50 |
| U6 (Forward) | miRNA | GAAGGATGACACGCAAATTCG | 21 |
