# Supplementary material for: Integrated Molecular and Phenotypic Profiling of Circulating Tumor Cells in Gastric Cancer: A Translational Perspective
Source: FASEB Bioadv. 2026 Aug 19;8(8):e70140. doi: 10.1096/fba.2025-00314 (PMC13489073; doi:10.1096/fba.2025-00314)
Supplement: Supplementary file 3 [file FBA2-8-e70140-s003.docx]

Supplementary Table 3. Differential Expression of miR-29b Across CTC Risk Groups

| CTC Group | Mean Expression (Â± SD) | N | Fold Change vs Normal | p-value vs Normal | p-value vs Intermediate | Significance |
| --- | --- | --- | --- | --- | --- | --- |
| Normal | 1.0000 Â± 0.2825 | 30 | Ref | Ref | - | - |
| Intermediate Risk | 0.2332 Â± 0.0324 | 18 | 0.233 | 0 | - | **** |
| High Risk | 0.0336 Â± 0.0029 | 4 | 0.034 | 0.00001 | 0.01257 | **** |
