# Supplementary material for: Integrated Molecular and Phenotypic Profiling of Circulating Tumor Cells in Gastric Cancer: A Translational Perspective
Source: FASEB Bioadv. 2026 Aug 19;8(8):e70140. doi: 10.1096/fba.2025-00314 (PMC13489073; doi:10.1096/fba.2025-00314)
Supplement: Supplementary file 4 [file FBA2-8-e70140-s004.docx]

Supplementary table 2. Statistical Test Results for CTC Changes Before and After Treatment

| **Phenotype** | **Test Type** | **Test Statistic** | **p-value** | **Sample Size (n)** |
| --- | --- | --- | --- | --- |
| CTC Epithelial | Paired t-test | 2.42 | 0.0246 | 22 |
| CTC Epithelial | Wilcoxon signed-rank | 9 | 0.0258 | 22 |
| CTC Mesenchymal | Paired t-test | 3.03 | 0.0064 | 22 |
| CTC Mesenchymal | Wilcoxon signed-rank | 3.5 | 0.0002 | 22 |
